# Benefits of Massage-Myofascial Release Therapy on Pain, Anxiety, Quality of Sleep, Depression, and Quality of Life in Patients with Fibromyalgia

**DOI:** 10.1155/2011/561753

**Published:** 2010-12-28

**Authors:** Adelaida María Castro-Sánchez, Guillermo A. Matarán-Peñarrocha, José Granero-Molina, Gabriel Aguilera-Manrique, José Manuel Quesada-Rubio, Carmen Moreno-Lorenzo

**Affiliations:** ^1^Department of Nursing and Physical Therapy, University of Almería (UAL), 04120 Almería, Spain; ^2^Health Distric of La Vega-Andalusian, Health Public Service (Málaga), 29200 Málaga, Spain; ^3^Department of Statistic, University of Granada (UGR), 18071 Granada, Spain; ^4^Department of Physical Therapy, University of Granada (UGR), 18071 Granada, Spain

## Abstract

Fibromyalgia is a chronic syndrome characterized by generalized pain, joint rigidity, intense fatigue, sleep alterations, headache, spastic colon, craniomandibular dysfunction, anxiety, and depression. The purpose of the present study was to determine whether massage-myofascial release therapy can improve pain, anxiety, quality of sleep, depression, and quality of life in patients with fibromyalgia. A randomized controlled clinical trial was performed. Seventy-four fibromyalgia patients were randomly assigned to experimental (massage-myofascial release therapy) and placebo (sham treatment with disconnected magnotherapy device) groups. The intervention period was 20 weeks. Pain, anxiety, quality of sleep, depression, and quality of life were determined at baseline, after the last treatment session, and at 1 month and 6 months. Immediately after treatment and at 1 month, anxiety levels, quality of sleep, pain, and quality of life were improved in the experimental group over the placebo group. However, at 6 months postintervention, there were only significant differences in the quality of sleep index. Myofascial release techniques improved pain and quality of life in patients with fibromyalgia.

## 1. Introduction

Fibromyalgia (FMS) is a chronic syndrome characterized by generalized pain, joint rigidity, and intense fatigue. Other frequently associated symptoms are sleep alterations, headache, spastic colon, craniomandibular dysfunction, anxiety, and depression. Fibromyalgia has a negative effect on the quality of life of patients, who often feel incapable of performing such basic daily life activities as walking, going up stairs, or lifting objects, increasing their disability index and utilization of health services [1, 2].

The etiology of the disease is currently unknown but several hypotheses have been developed, given that fibromyalgia syndrome is a multidisciplinary problem approached from different perspectives. Histological and histochemical studies have demonstrated that it is not an inflammatory process [3]. The most widely accepted hypothesis is that chronic pain in FMS is of muscle origin, although plasma muscle enzyme levels, electromyographic studies, and muscle biopsies have proven completely normal [4–6]. The methodological approach to muscle studies has been varied, from muscle biopsies for structural study to electromyograms and muscle metabolism studies using spectroscopic nuclear magnetic resonance (NMR). Results have shown characteristics associated with pain perception changes, sleep alterations, decrease in brain serotonin levels, and abnormalities in microcirculation and muscle energy metabolism [7]. Taken together, these alterations contribute to neuronal hyperreactivity and myofascial distress, indicating that the origin of the pain may be related to myofascial trigger points or musculoskeletal changes. 

 Modifications of adrenocorticotropic hormone levels and a decrease in plasma serotonin have been reported in some of these patients, indicating central nervous system (CNS) involvement and neurohormonal axis changes. In this regard, there is evidence of interaction between low sleep quality and low plasma levels of serotonin, a neurotransmitter that functions in the neuromodulation of sleep, pain, and mood [8–10]. Various studies have demonstrated that the perception of pain in FMS is related to CNS modifications that translate into the amplification of nociceptive impulses [11]. This phenomenon is designated “central sensitization” and is believed to result from the plasticity of neuronal synapses in response to previous painful experiences. Different degrees of central sensitization have been described, explaining the variations in pain reported by FMS patients. Although there is no specific peripheral tissue anatomy that characterizes fibromyalgia, this does not reduce the importance of peripheral nociceptive mechanisms [12, 13]. CNS sensitization leads peripheral pain generators to trigger major nociceptive impulses that will in turn increase central sensitization. The most frequent peripheral pain generators in FMS include: myofascial trigger points, degenerative joint disease, inflammatory joint disease, bursitis, tendinitis, development alterations, hypermobility syndrome, neuropathic pain, injuries, traumas, repeated muscle pulls, visceral pain, disk herniation, spinal stenosis, and recurrent cephalalgia [14, 15].

 There is no evidence of muscle disease in FMS but there are reports of dysfunction in intramuscular connective tissue or fascia; fascial inflammation triggers a peripheral nociceptive stimulus that leads to central sensitization in FMS [16–18]. Immunohistochemical studies of fascial tissue biopsies reveal an increase in collagen levels and inflammation mediators in connective tissue surrounding muscle cells [16]. In line with these findings, an exploratory and tentative study suggested the presence of latent and active myofascial trigger points in patients with FMS and myofascial pain syndrome [17–20].

### 1.1. Purpose of the Study

Because the cause of FMS syndrome remains unknown, treatment is usually in response to symptoms. However, the effectiveness of pharmacological and nonpharmacological treatments has been limited. The purpose of this study was to determine the benefits of massage-myofascial release therapy on pain, anxiety, quality of sleep, depression, and quality of life in patients with FMS.

## 2. Methods

### 2.1. Study Design

A randomized controlled trial was performed, with placebo group (FMS patients subjected to a sham protocol of magnotherapy) and experimental group (FMS patients who received massage-myofascial release therapy). The study period was from January 1 2009 to January 31 2010.

### 2.2. Setting and Patients

We recruited patients diagnosed with FMS (by clinicians according to the criteria of the American College of Rheumatology) [21] who belong to the Almeria Fibromyalgia Association (AFIAL-Spain). Inclusion criteria were FMS diagnosis, age from 18 to 65 years (working age range), no regular physical activity, and agreement to attend evening therapy sessions. Exclusion criteria were: nonagreement to study participation, receipt of other nonpharmacologic therapies, presence of cardiac, renal or hepatic insufficiency, cardiovascular event during the previous year, and presence of peripheral arterial or venous insufficiency, physical or psychological disease, infection, fever, hypotension, respiratory alterations limiting treatment application, skin integrity alterations, and failure to comply with prescribed pharmaceutical therapy [22].

 Out of the 231 accessible patients with a medical diagnosis of FMS, 35 were unable to participate due to incompatibility with their work schedule and the remaining 196 were subjected to a randomization process to select a sample of 100 patients; 36 of these did not meet study inclusion criteria, and the remaining 64 were randomly assigned to an experimental (*n*  =  32) or placebo (*n*  =  32) group by using sealed envelopes. Informed consent of patients was obtained, fulfilling the ethical criteria established in the Helsinki Declaration, modified in 2000, for research projects. In Spain, the current legislation for clinical trials is gathered in *Real Decreto 223/2004* of February 6. The study was approved by the ethics and research committee of the University of Almeria.

### 2.3. Measures

The instruments listed below were used to measure pain, anxiety, depression, quality of sleep, and quality of life. 

 Pain was assessed with the Visual Analog Scale (VAS), which assesses the pain intensity and degree of relief experienced by the patient (score of 0 = no pain; 10 = unbearable pain). Painful sensitive points were assessed by means of a pressure algometer (Wagner FPI 10-USA), exerting a pressure of 4 kg. This instrument consists of a sphere, on which the pressure measurements (10 levels of 0.5 Kg each) are shown, with a rubber end for exertion of the pressure. The 18 painful sensitive points described by the American College of Rheumatology were evaluated [23]. The index of internal consistency is 0.56–0.88 for the measurement of the pain VAS [24].

 Anxiety levels were determined with the 40-item State-Trait Anxiety Inventory (STAI), which measures anxiety as a stable dimension of personality (trait or tendency to anxiety) and also includes a state subscale to detect anxiety behaviors. Subjects report their feelings in general for the trait scale and how they feel at the time of questionnaire completion for the state anxiety scale. The state anxiety scale indicates the feelings or sensations of anxiety (not at all, somewhat, moderately so, very much so) at a specific moment in time. The trait anxiety scale indicates the frequency with which anxiety is experienced (almost never, sometimes, often, almost always). Factorial analyses identified four factors related to the presence or absence of anxiety in each scale: presence of state anxiety, absence of state anxiety, presence of trait anxiety, and absence of trait anxiety. The index of internal consistency is 0.90–0.93 for the measurement of state anxiety and 0.84–0.91 for trait anxiety [25].

The state of depression was determined with the Beck Depression Inventory (BDI), a self-applied 21-item questionnaire that assesses a wide spectrum of depressive symptoms. It focuses on the cognitive components of depression, which represent around 50% of the total questionnaire score. Out of the 21 items, 15 refer to ecological-cognitive symptoms and 6 to somatic-vegetative symptoms; each item has four response options in order of increasing symptom severity [26]. The aim of the questionnaire is to quantify symptoms rather than yield a diagnosis. The total questionnaire score ranges from 0 to 63 points, and the usual classifications are as follows: no depression: 0–9 points; mild depression: 10–18 points; moderate depression: 19–29 points; severe depression: ≥30 points. The reliability of the BDI is 0.65–0.72 and the Chronbach's alpha coefficient is 0.82 [27]. 

 The Pittsburgh Quality of Sleep Index Questionnaire (PSQI) was used to study the quality of sleep. It comprises 24 items; the subject responds to 19 of these items, and an individual living in the same dwelling (or hospital room) responds to the remaining 5. Scores are obtained on each of seven components of sleep quality: subjective quality, sleep latency, sleep duration, habitual sleep efficiency, sleep perturbations, use of hypnotic medication, and daily dysfunction. Each component is scored from 0 to 3 (0 = no problems; 3 = severe problems). Hence the total score (adding component scores) ranges from 0 to 21. The reliability coefficient of the PSQI is 0.78 [28, 29]. 

 Quality of life was assessed with the 36-item SF-36 Quality of Life Questionnaire on functional state, emotional well-being, and general health. Functional state is represented by the dimensions of physical function (10 items), social function (2 items), role limitations due to physical problems (4 items), and role limitations due to emotional problems (3 items). Emotional well-being includes mental health (5 items), vitality (4 items), and pain (2 items). Finally, perception of general health (5 items) and changes in health over time (1 item, not included in the final score) were assessed. For each dimension of the SF-36, items were codified, aggregated, and transformed into a scale ranging from 0 (worst health status) to 100 (optimal health status). Thus, a higher score in the different dimensions indicate a better health status and/or quality of life. The Chronbach's alpha coefficient for this questionnaire is 0.78–0.96 [30].

### 2.4. Procedure

The experimental group was formed by 30 patients and the control group by 29. In all subjects, pain, anxiety, depression, quality of sleep, and quality of life scores were determined before (baseline) and immediately after the 20-week intervention and again at one month and 6 months. 

#### 2.4.1. Intervention

The experimental group underwent a protocol of massage-myofascial release therapy during a weekly 90-minute session for 20 weeks. The treatment was applied by a physiotherapist specialized in massage-myofascial therapy and aimed to release myofascial restrictions at the sites of the 18 painful points reported by the American College of Rheumatology. The protocol was as follows: massage-myofascial release at insertion of the temporal muscle, release of falx cerebri by frontal lift, release of tentorium cerebelli by synchronization of temporals, assisted release of cervical fascia, release of anterior thoracic wall, release of pectoral region, lumbosacral decompression, release of gluteal fascia, transversal sliding of wrist flexors and fingers, and release of quadriceps fascia [31].

 The placebo group underwent a weekly 30-minute session of disconnected magnetotherapy for 20 weeks. With the patient in prone position, magnotherapy was applied on the cervical area (15 min) and lumbar area (15 min). Placebo group patients were unaware that they were receiving a sham treatment.

### 2.5. Data Analysis

SPSS version 18.0 was used for the data analyses. After a descriptive study of the demographic variables, the normal distribution of variables was examined by means of the Kolmogorov-Smirnov test. We calculated an imputed score for standardized scales missing ≤10% of responses. Independent *t*-tests were used to compare baseline demographic characteristics between participants and drop-outs and between the experimental and placebo groups (randomization test).

 Changes in scores for anxiety, pain, depression, and quality of life were analyzed by using a 2 (Groups: experimental and placebo) ×4 (Time points: baseline, immediately postintervention, at 1 and 6 months) repeated-measures analysis of variance (ANOVA). A Student's *t*-test for paired measures was used to determine the effectiveness of treatments. Differences between study groups were analyzed with a Student's *t-*test for independent samples. *P* < .05 was considered significant in all tests.

## 3. Results

**Figure 1:**
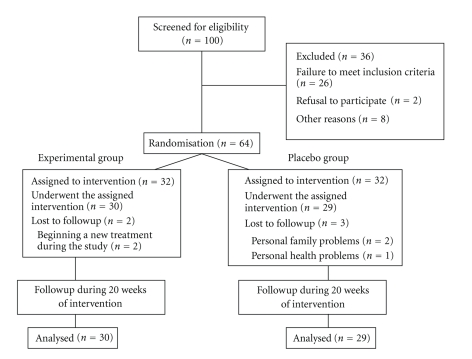
Follow of participants in the study.

Out of the sixty-four patients enrolled in the study, two were lost from the experimental group for starting another treatment and three dropped out of the control group due to family and personal problems (Figure 1). Therefore, the study was completed by 30 patients in the experimental group and 29 in the control group. At baseline, the two groups did not significantly differ in: demographic characteristics (Table 1), mean VAS score for pain (*P* < .087), trait anxiety (*P* < .074), state anxiety (*P* < .064), BDI (*P* < .081), sensitive points, any SF-36 dimension except for emotional role (*P* < .049), or any dimension of the PSQI except for sleep duration (*P* < .047).

### 3.1. Immediately after 20-Week Intervention

**Figure 2:**
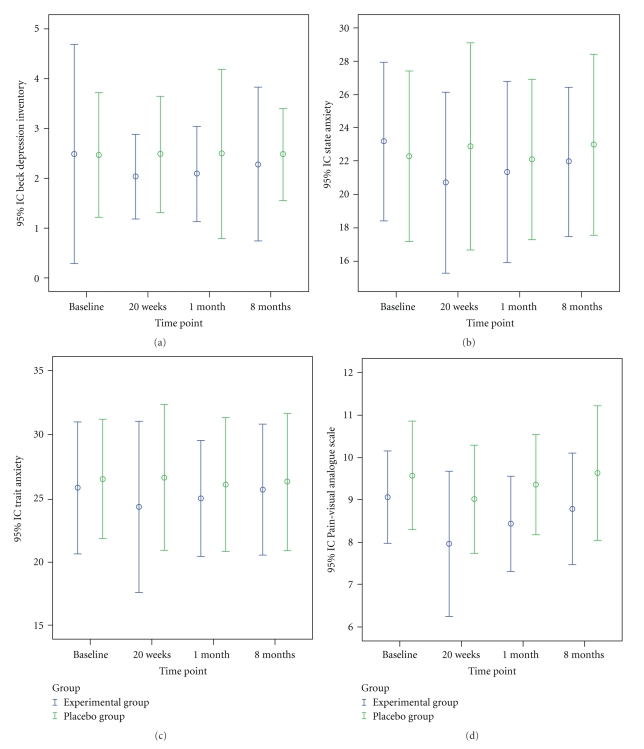
Comparison between study groups in levels of depression, anxiety, and pain. Values are presented as means with errors bars.

Postintervention, the experimental group showed a significant improvement in VAS score for pain (*P* < .043,) *versus* baseline and in comparison to the control group (Figure 2). There were significant reductions in sensitive points as measured by pressure algometer at left lower cervicals (*P* < .023), right gluteal muscle (*P* < .038), left gluteal muscle (*P* < .043) and right greater trochanter (*P* < .039). No changes were observed in the placebo group. Tables 2 and 3 show the significant differences between the groups.

 The experimental group also showed a significant improvement in trait anxiety (*P* < .041) *versus* baseline and in comparison to the placebo group. There were no differences in state anxiety or BDI *versus* baseline or between groups (Figure 2). Among the SF-36 dimensions, the experimental group showed significant improvements in physical function (*P* < .007), physical role (*P* < .039), body pain (*P* < .043), and social function (*P* < .048) *versus* baseline.  Table 4 shows the significant differences between groups. No changes were observed in the placebo group. The experimental group showed a significant improvement *versus* baseline in sleep latency (*P* < .041) and sleep duration (*P* < .039) in the PSQI, while no changes were observed in the placebo group.  Table 5 shows the significant differences between the groups.

### 3.2. One Month Postintervention

One month postintervention, the experimental group showed a significant improvement in VAS score for pain (*P* < .043) *versus* baseline and in comparison to the control group (Figure 2) and a significant reduction *versus* baseline in painful sensitive points at left lower cervicals (*P* < .031), right gluteal muscle (*P* < .039), and right greater trochanter (*P* < .044). No changes were observed in the placebo group.

 The experimental group showed a significant improvement in trait anxiety (*P* < .043) but no significant differences were found between groups (Figure 2). No differences were observed in BDI score versus baseline or between groups. For the SF-36 dimensions, there were significant improvements in physical function (*P* < .01), while no changes were observed in the placebo group.  Table 4 shows the significant differences between the groups. For the PSQI items, the experimental group showed a significant improvement in sleep duration *versus* baseline (*P* < .045), while no changes were observed in the placebo group.  Table 6 shows the significant differences between groups. In the experimental group, repeated-measures ANOVA showed a significant time × groups interaction for: VAS score for pain (*F* = 0.396; *P* < .047), trait anxiety (*F* = 0.403; *P* < .045), physical function (*F* = 0.771; *P* < .028), physical role (*F* = 0.422; *P* < .044), and body pain (*F* = 0.633; *P* < .047) dimensions of the SF-36.

### 3.3. Six Months Postintervention

At 6 months postintervention, the experimental group showed a significant improvement versus baseline (*P* < .047) in sleep duration (PSQI) and for a tender point at right greater trochanter (*P* < .048). Tables 3 and 6 show the significant differences between the groups. No changes were found in the placebo group.

## 4. Discussion

In this study, a 20-week massage-myofascial release program significantly improved the pain, anxiety, quality of sleep, and quality of life in FMS patients. The treatment reduced the sensitivity to pain at sensitive points, mainly at the lower cervicals, gluteal muscles, and right greater trochanter. 

Fascial entrapment patterns can appear when a body segment stops receiving appropriate stimuli, establishing a pathological process with deficient circulation and limitation in nutrient supply to the fundamental substance of connective tissue, with its consequent densification. Because dense tissue is hypomobile, this situation leads to movement limitations [31]. Fat accumulation is therefore favored in the affected body segment, altering the properties of the connective tissue and perpetuating the dysfunction if not corrected. Areas of myofascial entrapment are highly sensitive and painful to all type of stimuli [32].

In the present study, massage-myofascial release therapy produced no changes in BDI scores. A follow-up period of more than six months is probably necessary for a more exhaustive analysis of the bidirectional relationship between pain and depression in FMS. The presence of depression may facilitate the expression of active trigger points and *viceversa *[17, 18]. Multidisciplinary approaches have achieved significant improvements in depression in FMS patients, attributed to the synergistic action of the different therapies against pain [33–37]. The comorbidity of pain and depression may be linked to central sensitization, in that the persistence of chronic pain and depression may indicate a common pathogenic mechanism attributable to alterations of the hypothalamus-hypophyseal-adrenal axis [38].

The significant improvements in SF-36 dimensions, physical function, and body pain observed after myofascial release therapy were also reported after aerobic exercise and multidisciplinary interventions in FMS patients [39–44]. These studies underlined the importance of motivation and reinforcement through health education to reduce body pain and improve quality of life perception. We could find no published reports of a significant improvement in state anxiety through the application of manual therapy alone, and this was also true in the present study. However, a multimodal approach has yielded improvements in state anxiety levels [45, 46], which may be explained by their positive impact on psychoemotional factors [47]. Anxiety and stress can affect proteoglycan synthesis and metabolism, hence interfering with the mechanical properties of connective tissue. If perpetuation of this phenomenon is combined with immobility phenomenon, fascial entrapment areas appear, triggering the emergence of painful points [48]. The improvements obtained in the PSQI by applying myofascial release techniques were similar to those obtained by the manipulation of conjunctive tissue [49]. The release of fascial restrictions, correcting visceral fascial dysfunction at intestinal level, may facilitate sleep by favoring the secretion of serotonin by platelets. A large number of these patients have intestinal problems or diseases, which may be due to neuroendocrine disorders that can affect serotonin secretion [50].

 One study limitation is the exclusion of 35 of the 231 eligible participants due to incompatibility with their work schedules. A further limitation is that patients with less severe pain may have been able to improve more rapidly.

## 5. Conclusions

This study demonstrated that massage-myofascial release therapy reduces the sensitivity to pain at tender points in patients with fibromyalgia, improving their pain perception. Release of fascial restrictions in these patients also reduces anxiety levels and improves sleep quality, physical function, and physical role. Massage-myofascial program can be considered as an alternative and complementary therapy that can achieve transient improvements in the symptoms of these patients.

## Figures and Tables

**Table 1:** Demographic characteristics of the groups.

Characteristics	Experimental (*n* = 30)	Placebo (*n* = 29)	*P*
Mean Age (SD)	49.32 (11.63)	46.29 (12.29)	.058
Sex (%)			
Female	94.36	96.42	.321
Male	5.64	3.58	.109
Educational level (%)			
No school	22 (73.3)	19 (65.5)	.131
Primary school	4 (13.3)	7 (24.1)	.122
Secondary school	3 (10)	1 (3.4)	.314
University school	1 (3.3)	2 (6.9)	.629
Work activity (%)			
Full-time	12 (40)	9 (31.03)	.119
Part-time	7 (23.3)	10 (34.5)	.107
Sick leave	2 (6.7)	4 (13.8)	.231
Unemployed	9 (30)	6 (20.7)	.116

*P*-value  < .05 between experimental and placebo groups.

**Table 2:** Differences between groups in numbers of patients with painful tender points (9 tender points I).

	Baseline PTP		*P*	20 Weeks PTP		*P*	1 Month PTP		*P*	6 Months PTP		*P*
Tender points	EG	PG		EG	PG		EG	PG		EG	PG	
RO	15	22	.173	9	20	.048*	11	22	.042*	13	21	.098
LO	13	21	.182	8	22	.035*	10	23	.032*	12	23	.044*
LCR	19	15	.058	13	16	.683	14	16	.732	16	18	.792
LCL	15	23	.100	5	22	.012*	7	21	.023*	11	20	.054
RTM	13	22	.101	9	21	.037*	12	23	.049*	12	22	.049*
LTM	18	17	.573	10	19	.058	13	23	.051	13	22	.050
RSM	11	20	.063	6	18	.345	7	17	.056	10	18	.108
LSM	14	18	.792	10	17	.108	12	19	.228	15	20	.677
2nd RR	16	13	.681	8	14	.330	13	15	.796	16	14	.255

*P-value  < .05. Values are presented as numbers of patients with painful tender points. Abbreviations: PTP: painful tender points; EG: experimental group; PG: placebo group; RO: right occiput; LO: left occiput; LCR: lower cervicals (righ-side); LCL: lower cerivicals (left-side); RTM: right trapezius muscle; LTM: left trapezius muscle; RSM: right supraspinatus muscle; LSM: left supraspinatus muscle; 2nd RR: second right rib.

**Table 3:** Differences between groups in numbers of patients with painful tender points (9 tender points II).

	Baseline PTP		*P*	20 Weeks PTP		*P*	1 Month PTP		*P*	6 Months PTP		*P*
Tender points	EG	PG		EG	PG		EG	PG		EG	PG	
2nd LR	19	17	.110	9	18	.059	11	20	.058	11	19	.103
RLE	13	21	.098	8	22	.051	12	21	.051	13	22	.063
LLE	11	18	.227	6	19	.032*	9	20	.046*	10	19	.066
RG	18	16	.110	6	17	.048*	8	19	.048*	12	18	.353
LG	17	18	.638	7	19	.034*	9	19	.059	9	20	.051
RGT	12	17	.680	3	16	.033*	6	18	.046*	8	18	.048*
LGT	9	14	.642	4	14	.057	6	16	.058	8	15	.168
RK	14	19	.638	11	20	.063	13	20	.173	14	19	.638
LK	13	20	.173	10	19	.068	11	20	.063	12	21	.058

*P-value  < .05. Values are presented as numbers of patients with painful tender points. Abbreviations: PTP: painful tender points; EG: experimental group; PG: placebo group; 2nd LR second left rib; RLE: right lateral epicondyle; LLE: left lateral epicondyle; RG: right gluteal muscle; LG: left gluteal muscle; RGT: right greater trochanter: LGT: left greater trochanter; RK: right knee; LK: left knee.

**Table 4:** Differences in quality of life (SF-36 questionnaire) between study groups.

	Baseline M (SD)		*P*	20 Weeks M (SD)		*P*	1 Months M (SD)		*P*	6 Months M (SD)		*P*
SF-36	EG	PG		EG	PG		EG	PG		EG	PG	
PF	5.23 (5.36)	50.24 (8.47)	.103	46.72 (6.71)	51.03 (8.24)	.012*	46.84 (7.22)	49.56 (9.32)	.049*	48.20 (7.43)	51.19 (6.32)	.281
PR	25.97 (7.32)	26.36 (6.25)	.553	22.91 (7.15)	26.32 (6.29)	.026*	24.64 (9.46)	28.97 (8.31)	.047*	25.49 (8.41)	27.53 (6.25)	.213
BP	76.56 (6.31)	78.93 (11.43)	.196	73.93 (8.21)	77.54 (11.63)	.040*	75.05 (7.21)	89.93 (14.63)	.046*	75.63 (8.22)	77.84 (9.66)	.293
GH	67.82 (5.21)	68.78 (7.22)	.203	65.20 (5.43)	69.85 (6.24)	.055	66.81 (6.19)	68.43 (8.21)	.093	67.53 (7.24)	68.13 (6.44)	.401
V	60.85 (6.41)	59.42 (5.32)	.301	63.53 (8.17)	59.99 (9.41)	.051	61.64 (8.66)	59.22 (6.25)	.055	62.15 (9.32)	58.93 (7.65)	.312
SF	64.03 (8.03)	64.43 (13.22)	.639	59.55 (4.22)	64.03 (10.15)	.028*	60.63 (10.81)	63.56 (9.97)	.081	61.27 (7.53)	63.96 (9.71)	.088
ER	48.98 (8.13)	46.55 (7.32)	.049*	46.42 (11.32)	47.74 (9.26)	.292	50.45 (7.23)	47.02 (6.43)	.057	49.11 (7.33)	46.90 (9.38)	.219
MH	77.45 (12.31)	81.10 (1.29)	.101	78.27 (10.22)	82.02 (11.67)	.074	75.01 (11.13)	78.34 (9.46)	.082	76.46 (10.12)	80.03 (12.43)	.126

*P-value  = .05. Values are presented as means and standard deviations (SD). Abbreviations: EG: experimental group; PG: placebo group; PF: physical function; PR: physical role; BP: body pain; GH: general health; V: vitality; SF: social function; ER: emotional role; MH: mental health.

**Table 5:** Differences between study groups in Pittsburgh sleep quality index score at baseline and after therapy.

	Baseline (*n* = number of patients)							20 Weeks (*n* = number of patients)						
	EG			PG			*P*	EG			PG			*P*
PSQI	NP	MP	SP	NP	MP	SP		NP	MP	SP	NP	MP	SP	
PSQ	0	6	24	1	9	19	.072	0	5	25	3	14	12	.052
SL	1	1	28	1	3	25	.836	1	16	13	2	6	21	.045*
SD	0	7	23	1	1	27	.047*	0	14	16	0	4	25	.041*
HSE	1	9	20	0	5	24	.321	0	15	15	0	7	22	.073
SDI	0	11	19	2	10	17	.223	0	22	8	2	6	21	.051
DD	0	28	2	0	26	3	.493	2	26	2	0	23	6	.082

*P-value = .05. Values are shown as *n* = number of patients with no problems, moderate problems, severe problems. Abbreviations: PSQI: Pittsburgh sleep quality index; EG: experimental group; PG: placebo group; PSQ: Pittsburgh subjective quality; SL: sleep latency; SD: sleep duration; HSE: habitual sleep efficiency; SDI: sleep disturbance; DD: daily dysfunction; NP: no problems; MP: moderate problems; SP: severe problems.

**Table 6:** Differences between study groups in Pittsburgh sleep quality index at 1 months and 6 months after treatments.

	1 Months (*n* = number of patients)							6 Months (*n* = number of patients)						
	EG			PG			*P*	EG			PG			*P*
PSQI	NP	MP	SP	NP	MP	SP		NP	MP	SP	NP	MP	SP	
PSQ	4	8	18	6	14	9	.106	5	5	20	6	6	7	.061
SL	3	13	14	1	10	18	.122	3	12	15	3	8	18	.213
SD	1	11	18	2	3	24	.041*	2	11	17	2	0	27	.047*
HSE	3	14	13	1	8	20	.051	1	15	14	1	6	22	.058
SDI	0	15	15	3	5	21	.054	0	14	16	3	5	21	.117
DD	5	16	9	2	17	10	.305	4	12	14	2	21	6	.053

*P-value  =  .05. Values are shown as *n* = number of patients with no problems, moderate problems, severe problems. Abbreviations: PSQI: Pittsburgh sleep quality index; EG: experimental group; PG: placebo group; PSQ: Pittsburgh subjective quality; SL: sleep latency; SD: sleep duration; HSE: habitual sleep efficiency; SDI: sleep disturbance; DD: daily dysfunction; NP: does not present problems; MP: moderate problems; SP: severe problems.
